# Widespread and widely widening? Examining absolute socioeconomic health inequalities in northern Sweden across twelve health indicators

**DOI:** 10.1186/s12939-019-1100-5

**Published:** 2019-12-18

**Authors:** Kinza Degerlund Maldi, Miguel San Sebastian, Per E. Gustafsson, Frida Jonsson

**Affiliations:** Department of Epidemiology and Global Health, Umeå University, SE-901 85 Umeå, Sweden

**Keywords:** Socioeconomic inequalities in health, Outcome-wide approach, Slope index of inequality, Time trends, Northern Sweden

## Abstract

**Background:**

Socioeconomic inequalities in health is a widely studied topic. However, epidemiological research tends to focus on one or a few outcomes conditioned on one indicator, overlooking the fact that health inequalities can vary depending on the outcome studied and the indicator used. To bridge this gap, this study aims to provide a comprehensive picture of the patterns of socioeconomic health inequalities in Northern Sweden over time, across a range of health outcomes, using an ‘outcome-wide’ epidemiological approach.

**Method:**

Cross-sectional data from three waves of the ‘Health on Equal Terms’ survey, distributed in 2006, 2010 and 2014 were used. Firstly, socioeconomic inequalities by income and education for twelve outcomes (self-rated health, self-rated dental health, overweight, hypertension, diabetes, long-term illness, stress, depression, psychological distress, smoking, risky alcohol consumption, and physical inactivity) were examined by calculating the Slope Index of Inequality. Secondly, time trends for each outcome and socioeconomic indicator were estimated.

**Results:**

Income inequalities increased for psychological distress and physical inactivity in men as well as for self-rated health, overweight, hypertension, long-term illness, and smoking among women. Educational inequalities increased for hypertension, long-term illness, and stress (the latter favouring lower education) in women. The only instance of decreasing income inequalities was seen for long-term illness in men, while education inequalities decreased for long-term illness in men and poor self-rated health, poor self-rated dental health, and smoking in women.

**Conclusion:**

Patterns of absolute socioeconomic inequalities in health vary by health and socioeconomic indicator, as well as between men and women. Overall, trends appear more stagnant in men while they fluctuate in women. Income inequalities seem to be generally greater than educational inequalities when looking across several different health indicators, a message that can only be derived from this type of outcome-wide study. These disparate findings suggest that generalised and universal statements about the development of health inequalities can be too simplistic and potentially misleading. Nonetheless, despite inequalities being complex, they do exist and tend to increase. Thus, an outcome-wide approach is a valuable method which should be utilised to generate evidence for prioritisations of policy decisions.

## Introduction

Socioeconomic inequalities in health – such as by income, education, or occupation – represent a major challenge for public health policy and practice in Sweden [1], Europe [2], and globally [3]. This concept is important because it focuses on systematic and often deemed unfair differences in health between population sub-groups, who by some socioeconomic indicators are considered more or less disadvantaged [4].

Health inequalities have been thoroughly studied and observed for a wide range of health outcomes, such as weight gain, overweight, obesity [5, 6], self-rated health [7], smoking [7], mental health problems [8], hypertension and diabetes [9, 10]. However, the body of literature studying the disparities is spread across countless studies which typically use one or several socioeconomic indicators while focusing on only one or a few health outcomes. The fact that health inequalities could vary depending on the socioeconomic indicator and health outcome studied, thereby being potentially expressed in different and sometimes conflicting ways, has so far only been sparsely studied. For example, socioeconomically disadvantaged groups usually report poorer general and mental health than the most advantaged, while the reverse has been shown for risky alcohol use in France [11]. Along the same lines, while the disparities have widened for diabetes, they appear to have narrowed for obesity in the US and UK [9]. Similarly, in Sweden the socioeconomically better-off typically report better self-rated health than the worst-off, while the difference between the socioeconomic groups in psychological distress appear smaller [7].

Although health inequalities may be assessed through different indicators of socioeconomic position to capture the various aspects and dimensions of the phenomenon [12] in Sweden public health reports have typically focused mainly on health differences by education [13]. In this regard, subsequent writings have had a tendency to conclude that socioeconomic inequalities in health have increased over time [14], although studies examining differences by income indicate that the interpretation may not be as straight forward [15]. In addition, these reports [13, 14] typically present educational differences in health by sex, indicating that the patterns of socioeconomic inequalities in health may vary between women and men.

To gain a more nuanced picture of the complex health inequality panorama and to provide evidence that may be useful for prioritisation in policy decisions in Sweden, different socioeconomic indicators and a wide range of health outcomes may need to be simultaneously assessed. In this regard, VanderWeele [16] has proposed an ‘outcome-wide’ epidemiological approach where the association between a single predictor and multiple outcomes are tested, arguing that some exposures may influence different outcomes heterogeneously in beneficial or harmful ways.

Against this background, the current study intends to provide a comprehensive yet nuanced picture of the patterns of socioeconomic inequalities in health in Northern Sweden. The aim of the research was thus to examine trends in income and educational inequalities across a range of twelve health outcomes using an ‘outcome-wide’ epidemiological approach.

## Method

### Design and study population

This study used cross-sectional data, with a four-year interval, from the three most recent available waves of the ‘Health on Equal Terms’ survey distributed in 2006, 2010, and 2014, in the four northernmost counties of Sweden: Norrbotten, Västerbotten, Västernorrland, and Jämtland/Härjedalen [17]. The sample was selected using a two-step probabilistic sampling method which gave a weighted representative sample of the population aged 16–84 at municipal level. For this study, individuals aged 26–84 years were included based on the rationale that those below 26 might still be in education and not settled on the job market. The initial sample consisted of 27,771, 36,627 and 26,646 individuals in 2006, 2010 and 2014, respectively. After excluding participant below age 26, the analytical sample size for the respective years was 23,448, 33,327 and 22,637 individuals. In this study we only work with coded data (pseudo-ID) where personal information cannot be directly or indirectly tied to a specific individual.

### Operationalisation of variables

Based on the idea that socioeconomic inequalities could vary across different aspects of health as indicated by previous research, twelve self-reported outcomes grouped into four dimensions were identified: general health (self-rated health, self-rated dental health, overweight), physical health (hypertension, diabetes, long-term illness), mental health (stress, depression, psychological distress) and lifestyle behaviours (smoking, risky alcohol consumption, physical inactivity).

#### General health

*Self-rated health* (SRH) was operationalised as good or very good (= 0) and fair, poor, or very poor (= 1) general state of health. *Self-rated dental health* (SRDH) was ranked as good or very good (= 0) and neither good nor poor, quite poor, or very poor (= 1) dental health. Self-reported *overweight* was measured according to body mass index (BMI) as the ratio of weight to height with a BMI < 25 (= 0) and BMI > 25 (= 1).

#### Physical health

*Hypertension* and *diabetes* were operationalised as no hypertension or diabetes (= 0) and yes but with no discomfort, yes with minor discomfort, and yes with severe discomfort (= 1). *Long-term illness* was operationalised as no long-term illness (= 0) and long-term illness (= 1). All these variables were also self-reported.

#### Mental health

*Stress* was operationalised as experiencing none at all (= 0) and experiencing stress to some extent, quite a lot, and very much (= 1). *Depression* was ranked as not at all (= 0) and no more than usual, rather more than usual, and much more than usual (= 1). *Psychological distress* was measured using the GHQ-12 [18, 19], which is an instrument developed for non-psychotic mental illness where the participant answers if they have experienced symptoms and behaviours such as: being able to concentrate, enjoying day-to-day activities, feeling depressed, being unhappy, and being capable of making decisions. Each item assesses the severity of the symptoms on a four-point scale as ‘less than usual’, ‘no more than usual’, ‘rather more than usual’, or ‘much more than usual’. The items were recoded into more or less severity and then summed up into an index with a range of 0–12 and dichotomised, with a cut-off point of higher than two indicating distress.

#### Lifestyle habits

*Smoking* was operationalised as current daily smoker (= 1) or not (= 0). The variable *risky alcohol consumption* (referred to as alcohol use) was operationalised, according to the National Institute of Public Health [17], as consuming daily or almost every day, a few times a week, once a week, and 2–3 times a month (= 1), and once a month, once or a few times every 6 months, and less often or never (= 0). *Physical inactivity* (referred to as inactivity) was rated as sedentary leisure time (= 1) and moderate exercise in leisure time, moderate, regular exercise in leisure time, and regular exercise and training (= 0).

### Socioeconomic indicators

To assess socioeconomic inequalities in the above twelve health outcomes, we used the indicators of income and education. These were chosen to capture different dimensions or aspects of socioeconomic position, such as availability to materialistic resources and knowledge how to use resources [12] and because the Health on Equal Terms survey includes information on income and education from the Swedish registers, which is more reliable as compared e.g. to occupation, that is self-reported.

The education variable was classified according to the Statistics Sweden system as: ‘Compulsory education’ (= 5), ‘Secondary education up to 2 years’ (= 4), ‘Secondary education 3 years’ (= 3), ‘Post-secondary education less than 3 years’ (= 2), and ‘Post-secondary education 3 years or more’ (= 1). Compulsory school in Sweden corresponds to 9 years of primary and lower secondary school, secondary education to two or three additional years of voluntary school where students chose a field of study and post-secondary education to university and other forms of post-secondary education.

The income variable was based on annual disposable income for the individual, consisting of all taxable income, total earnings, income from business activities, property income, capital gains, pension, and debt. Income was divided into quintiles ranging from the richest 20% (= 1) to the poorest 20% (= 5). The information was retrieved from the tax registry using each individual’s Swedish Personal Identification Number.

### Data analysis

To provide a nuanced picture of the patterns and trends of socioeconomic inequalities in health, the data analysis consisted of three steps. Firstly, descriptive characteristics of the sample, indicators and the outcomes was estimated. Mean value for age and income, proportion of education level in the sample and proportion of poor outcome in the health variables was calculated. Secondly, to assess socioeconomic inequalities in the above health outcomes, the Slope Index of Inequality (SII) [20, 21] was estimated. SII is a regression-based summary measure recommended when making comparisons over time or across populations as it takes the whole socioeconomic distribution into account, rather than only comparing the two most extreme groups [22, 23]. To calculate SII, income and education were ranked from the highest to the lowest group. The population of each socioeconomic group covered a range in the cumulative distribution of the population and was given a ridit score which corresponded to the average cumulative frequency of the group [20, 21]. For example, in year 2006, women with post-secondary education 3 years or more contained 0.3% of the population, the range of individuals in this category is from 0.00 to 0.003, giving a mean of 0.0015, which is the value assigned to this category. The next education level, post-secondary education less than 3 years, consists of 27.14% of the population and is given the corresponding value of 0.1372 (0.0015 + [0.2714/2]) and so on [22, 24]. SII coefficients were obtained by generalized linear models, using binomial family and identity link functions, with the outcome regressed on the ridit scores, separately by each indicator and controlling for age [25]. The value of the β coefficient corresponds to the point estimate of SII, which can be interpreted as the estimated absolute prevalence difference of the outcome between the lowest and the highest socioeconomic group, taking into account the size and prevalence of all intermediate groups. Thirdly, for each outcome and socioeconomic indicator, time trends were estimated by adding a two-way interaction term ridit score × survey year (i.e 2006, 2010, 2014). STATA 13 software was used for the analysis. Patterns of socioeconomic inequalities in health can vary with age and between men and women, and it has been suggested that sex should be considered in the analysis [26, 27] thus all analyses were adjusted for age and stratified by sex.

An analysis was also carried out to assess how much the missingness varied between the outcomes. Missing data among the outcomes was relatively consistent, varying between 0.5 and 3.6%, except for three variables; diabetes, hypertension and alcohol use. Alcohol use was the variable with the most missing data; 17.1% in 2006, 19.2% in 2010 and 17.3% in 2014. The missingness of diabetes for the corresponding years was 7.9, 11.9 and 3.5% and for hypertension 4.3, 5.8 and 3%. Missing data was also consistent between the surveys, with an average of 4.1% in 2006, 4.5% in 2010 and 4.4% in 2014.

## Results

Table 1 shows the sociodemographic characteristics, such as age, socioeconomic position and prevalence of health outcomes, of the study populations by year and sex. The sample consisted of slightly more women than men, the mean age between the sexes was similar with men being slightly older than women. The results section below presents the prevalence of the health outcomes and the main findings organized by the four dimensions of outcomes (general health, physical health, mental health, and lifestyle habits). Unless otherwise stated, all income and educational inequalities in outcomes were to the disadvantage of the poorer or lower-educated group and all results are adjusted for age.

**Table 1:** Descriptive statistics for selected characteristics of participants of the Health on Equal Term survey according to sex: 2006, 2010 and 2014. Mean (standard deviation) for age and income, and N (proportions, %) for the remaining variables

Measures	Estimate					
	2006 (*n* = 23,448)		2010 (*n* = 33,327)		2014 (*n* = 22,637)	
	Men	Women	Men	Women	Men	Women
Control variables						
Age	54.81 (16.05)	54.59 (16.41)	57.42 (15.72)	56.12 (16.20)	57.68 (15.62)	56.24 (16.13)
Sex	10,983 (46.84)	12,465 (53.16)	15,442 (46.33)	17,885 (53.67)	10,568 (46.68)	12,069 (53.32)
Socioeconomic variables						
Income	174,956 (79,704)	144,334 (207,002)	218,792 (167,395)	178,795 (420,767)	241,612 (152,955)	191,920 (96,466)
Education						
Compulsory education	2265 (23.37)	1959 (17.94)	3525 (22.91)	3220 (18.06)	2194 (20.96)	1805 (15.04)
2 years secondary education	5159 (53.23)	5567 (50.97)	7696 (50.02)	8230 (46.15)	5545 (52.98)	5903 (49.17)
3 years secondary education	555 (5.73)	399 (3.65)	823 (5.35)	616 (3.45)	591 (5.65)	431 (3.59)
Post-secondary education < 3 years	1650 (17.0)	2964 (27.14)	3132 (20.35)	5647 (31.67)	2063 (19.71)	3816 (31.79)
Post-secondary education > 3 years	62 (0.64)	33 (0.30)	211 (1.37)	120 (0.67)	73 (0.70)	50 (0.42)
Health indicators						
General health						
Poor self-rated health	4038 (37.32)	5254 (34.96)	3575 (34.25)	4897 (40.01)	6258 (36.27)	4193 (35.31)
Poor self-rated dental health	3583 (33.17)	4492 (29.42)	3061 (29.88)	3263 (26.63)	4060 (23.02)	2571 (22.11)
Overweight	6862 (63.39)	9910 (65.16)	6783 (65.89)	6004 (49.64)	9027 (52.08)	6087 (52.41)
Physical health						
Hypertension	2751 (26.24)	4713 (32.57)	3547 (34.6)	3175 (26.58)	5208 (30.8)	3670 (31.33)
Diabetes	860 (8.49)	1432 (10.56)	1064 (10.41)	705 (6.15)	1131 (7.15)	754 (6.48)
Long-term illness	4882 (45.2)	6650 (43.72)	4617 (44.28)	5522 (45.36)	7589 (43.32)	5079 (42.84)
Mental health						
Stress	4561 (41.73)	5734 (37.6)	3723 (36.12)	6226 (50.19)	8033 (45.46)	5290 (45.24)
Depression	4528 (41.56)	6230 (40.88)	4157 (40.69)	5933 (47.96)	8077 (45.76)	5349 (46.13)
Psychological distress	1250 (11.38)	1956 (12.78)	1145 (10.83)	1968 (15.79)	3069 (17.35)	1796 (14.66)
Lifestyle habits						
Smoking	1215 (11.17)	1466 (9.73)	904 (8.64)	1799 (14.61)	1962 (11.23)	1146 (9.6)
Risky alcohol consumption	1091 (11.36)	1444 (10.96)	938 (10.28)	287 (2.92)	439 (3.19)	236 (2.47)
Physical inactivity	1533 (14.19)	2078 (13.7)	1401 (13.73)	1684 (13.8)	2460 (14.04)	1591 (13.71)

### General health

**Fig. 1:**
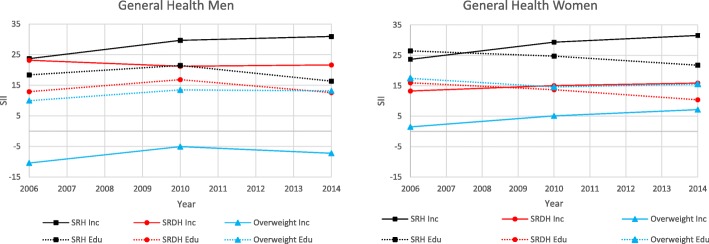
General Health. Slope index of inequality for participants in the Health on Equal Term survey according to sex, income and education: 2006, 2010 and 2014

The results for the general health dimension are presented below as well as in Fig. 1 and Table 2.

**Table 2:** General Health. Slope index of inequality for participants in the Health on Equal Term survey according to sex, income and education: 2006, 2010 and 2014

Outcome	Men			Women		
	2006	2010	2014	2006	2010	2014
SRH						
Income SII (CI)	27.55 (24.35, 30.75)	29.66 (27.01, 32.3)	30.91 (27.58, 34.24)	23.67 (20.51, 26.82)	29.33 (26.81, 31.86)	31.53 (28.42, 34.64)
P trend income	0.47			< 0.01		
Education SII (CI)	18.37 (14.69, 22.06)	21.44 (18.57, 24.31)	16.32 (12.85, 19.8)	26.46 (22.98, 29.95)	24.74 (21.99, 27.5)	21.74 (18.41, 25.08)
P trend education	0.64			0.02		
SRDH						
Income SII (CI)	23.14 (19.92, 26.36)	21.17 (18.56, 21.78)	21.6 (18.24, 24.96)	13.26 (10.35, 16.17)	15.03 (12.78, 17.29)	15.88 (13.04, 18.71)
P trend income	0.10			0.71		
Education SII (CI)	12.89 (9.16, 16.62)	16.81 (13.98, 19.64)	12.58 (9.07, 16.08)	15.97 (12.75, 19.18)	13.7 (11.22, 16.17)	10.38 (7.4, 3.37)
P trend education	0.42			< 0.01		
Overweight						
Income SII (CI)	−10.43 (−13.77, −7.08)	−5.08 (−7.86, −2.31)	−7.26 (−10.77, −3.75)	1.47 (−1.86, 4.79)	5.1 (2.35, 7.85)	7.14 (3.64, 10.64)
P trend income	0.07			0.05		
Education SII (CI)	9.94 (6.16, 13.73)	13.46 (10.51, 16.41)	13.16 (9.58, 16.74)	17.44 (13.74, 21.14)	14.6 (11.66, 17.54)	15.54 (11.96, 19.13)
P trend education	0.44			0.10		

^1^All estimates are age-adjusted

#### Self-reported health (SRH)

The prevalence of fair, poor, and very poor SRH decreased between each survey for both men and women. Both sexes experienced income inequalities in SRH of a similar magnitude. The income inequalities in women, but not in men, increased significantly over time. Education inequalities appeared larger among women compared with men in SRH. While men did not experience a significant change over time, a significant decrease was observed among women. In both sexes, income inequalities in SRH appeared larger compared with education inequalities.

#### Self-reported dental health (SRDH)

The prevalence of poor SRDH decreased overall for both men and women, but men experienced a slight increase in 2014. Income inequalities in SRDH appeared larger in men compared with women, but none experienced significant changes over time. Regarding education inequalities, the magnitude appeared similar among both sexes, with women experiencing a significant decrease over time in SRDH. Income inequalities appeared larger compared with education inequalities in men, whereas these were similar among women.

#### Overweight

The prevalence of overweight increased between each survey for both sexes. Men and women experienced a similar magnitude in income inequalities but in opposite directions: among men this favoured the low-income group. Women, but not men, experienced a significant increase in income inequalities over time in overweight. Education inequalities showed up as larger among women compared with men, with no significant changes over time for either sex. Both sexes appeared to experience larger education inequalities compared with income inequalities.

### Physical health

**Fig. 2:**
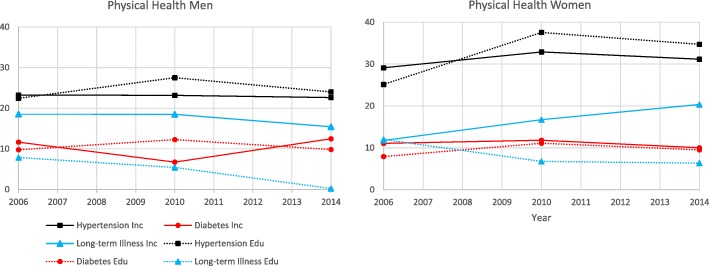
Physical Health. Slope index of inequality for participants in the Health on Equal Term survey according to sex, income and education: 2006, 2010 and 2014

The results for the physical health dimension are presented below as well as in Fig. 2 and Table 3.

**Table 3:** Physical Health. Slope index of inequality for participants in the Health on Equal Term survey according to sex, income and education: 2006, 2010 and 2014

Outcome	Men			Women		
	2006	2010	2014	2006	2010	2014
Hypertension						
Income SII (CI)	23.24 (20.31, 26.17)	23.15 (20.44, 25.86)	22.61 (19.12, 26.09)	29.12 (26.42, 31.81)	32.89 (30.47, 35.31)	31.16 (28.13, 34.19)
P trend income	0.16			< 0.01		
Education SII (CI)	22.43 (19.46, 25.4)	27.5 (24.77, 30.24)	24.00 (20.6, 27.41)	25.14 (22.38, 27.9)	37.56 (35.1, 40.03)	34.71 (31.69, 37.73)
P trend education	0.78			< 0.01		
Diabetes						
Income SII (CI)	11.64 (9.83, 13.44)	6.73 (5.22, 8.24)	12.47 (10.3, 14.64)	11.07 (9.66, 12.49)	11.81 (10.5, 13.12)	10.02 (8.68, 11.36)
P trend income	0.22			0.24		
Education SII (CI)	9.77 (7.98, 11.56)	12.26 (10.51, 14.02)	9.83 (7.72, 11.95)	7.93 (6.53, 9.34)	11.10 (9.66, 12.54)	9.51 (7.93, 11.09)
P trend education	0.99			0.49		
Long-term illness						
Income SII (CI)	18.53 (15.15, 21.91)	18.49 (15.66, 21.33)	15.42 (11.82, 19.01)	11.78 (8.52, 15.05)	16.72 (14.04, 19.41)	20.36 (16.99, 23.72)
P trend income	0.03			0.01		
Education SII (CI)	7.86 (3.97, 11.74)	5.43 (2.38, 8.47)	00.23 (−3.49, 3.95)	12.00 (8.32, 15.66)	6.76 (3.86, 9.65)	6.36 (2.84, 9.88)
P trend education	< 0.01			< 0.01		

^1^All estimates are age-adjusted

#### Hypertension

The prevalence of hypertension increased between each survey for both men and women. Women appeared to experience larger income inequalities compared with men. Significant changes over time were observed among women but not among men in income inequalities in hypertension. Similarly, education inequalities appeared larger among women than men, with a significant increase over time only among women. In both sexes, income and education inequalities were similar.

#### Diabetes

The prevalence of diabetes increased for men, with a peak in 2010, and remained fairly stable for women. The magnitude of income inequalities appeared similar between the sexes with neither experiencing significant changes over time. A similar pattern was observed in terms of educational inequalities in diabetes. Both sexes further appeared to experience similar inequalities in income and education.

#### Long-term illness

The prevalence of long-term illness decreased among women but remained fairly stable for men. The magnitude of income inequalities turned out to be similar in both sexes. However, while income inequalities significantly decreased for men over time, they increased for women. Education inequalities appeared larger among women compared with men, with both sexes experiencing a significant decrease over time. Income inequalities appeared larger than education inequalities in both sexes in long-term illness.

### Mental health

**Fig. 3:**
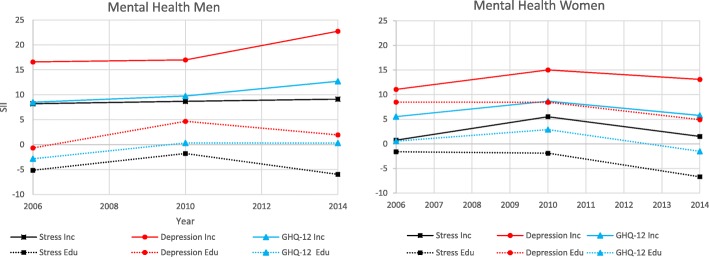
Mental Health. Slope index of inequality for participants in the Health on Equal Term survey according to sex, income and education: 2006, 2010 and 2014

The results for the mental health dimension are presented below as well as in Fig. 3 and Table 4.

**Table 4:** Mental Health. Slope index of inequality for participants in the Health on Equal Term survey according to sex, income and education: 2006, 2010 and 2014

	Men			Women		
	2006	2010	2014	2006	2010	2014
Stress						
Income SII (CI)	8.19 (4.85, 11.53)	8.67 (5.96, 11.38)	9.11 (5.67, 12.56)	0.75 (−2.49, 3.99)	5.51 (2.87, 8.16)	1.51 (−1.89, 4.9)
P trend income	0.42			0.53		
Education SII (CI)	−5.14 (−9.01, − 1.27)	− 1.79 (−4.74, 1.16)	−5.98 (−9.52, − 2.44)	− 1.61 (− 5.31, 2.09)	−1.9 (− 4.76, 0.96)	−6.69 (− 10.18, − 3.2)
P trend education	0.18			<.0.01		
Depression						
Income SII (CI)	16.58 (13.2, 19.96)	16.99 (14.15, 19.84)	22.74 (19.13, 26.35)	11.07 (7.79, 14.35)	15.02 (12.31, 17.73)	13.09 (9.6, 16.57)
P trend income	0.24			0.32		
Education SII (CI)	−0.66 (−5.57, 3.26)	4.67 (1.59, 7.75)	1.94 (−1.83, 5.71)	8.49 (4.76, 12.23)	8.46 (5.51, 11.4)	4.91 (1.27, 8.54)
P trend education	0.47			0.37		
Psychological distress						
Income SII (CI)	8.49 (6.47, 10.5)	9.74 (7.93, 11.55)	12.69 (10.67, 14.7)	5.53 (3.22, 7.84)	8.67 (6.67, 10.65)	5.77 (3.41, 8.12)
P trend income	0.01			0.92		
Education SII (CI)	−2.85 (−5.28, −0.41)	0.34 (−1.71, 2.4)	0.31 (−1.94, 2.56)	00.55 (−1.97, 3.08)	2.9 (00.76, 5.03)	−1.52 (−3.88, 0.84)
P trend education	0.77			0.01		

^1^All estimates are age-adjusted

#### Stress

The prevalence of stress decreased between each survey for both men and women. Income inequalities in men appeared larger compared with women, with the latter only having statistically significant results in 2010. Income inequalities did not significantly change over time in either of the sexes in stress. By contrast, the education inequalities favoured the less educated, with similar inequalities in both sexes. However, inequalities were only statistically significant among women in 2014 and among men in 2006 and 2014. While men did not experience statistically significant changes over time, educational inequalities increased among women in stress. Men appeared to experience larger inequalities in income compared with education, while these appeared similar among women.

#### Depression

The prevalence of depression appeared fairly stable over the years in both sexes. Men appeared to experience larger income inequalities in depression compared with women but no significant changes over time for either sex was found. In contrast, the magnitude of education inequalities appeared greater among women compared with men, with men only experiencing statistically significant results in 2010. Education inequalities did not statistically change over time in depression. Both sexes appeared to experience larger inequalities in income compared with education.

#### Psychological distress

Similar to depression, the prevalence of psychological distress remained relatively stable. Men appeared to experience larger income inequalities compared with women, with a statistical increase over time; this was not the case for women. Education inequalities were statistically significant for men in 2006 in favour of the lower educated and in 2010 for women in favour of the higher educated. Thus, men and women experienced similar magnitudes of inequalities in psychological distress but in opposite directions. The trend for women significantly increased over time. Both in men and women, income inequalities appeared larger compared with educational ones.

### Lifestyle habits

**Fig. 4:**
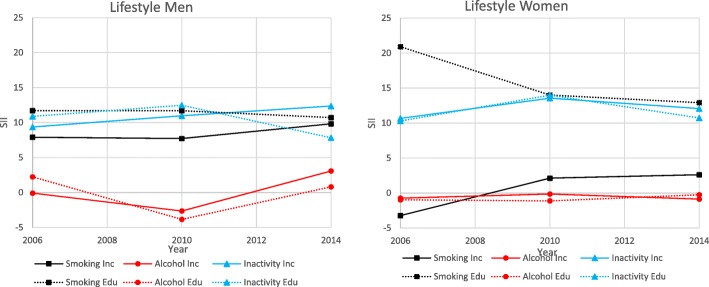
Lifestyle Habits. Slope index of inequality for participants in the Health on Equal Term survey according to sex, income and education: 2006, 2010 and 2014

The results for the lifestyle habits dimension are presented below as well as in Fig. 4 and Table 5.

**Table 5:** Lifestyle Habits. Slope index of inequality for participants in the Health on Equal Term survey according to sex, income and education: 2006, 2010 and 2014

Outcome	Men			Women		
	2006	2010	2014	2006	2010	2014
Smoking						
Income SII (CI)	7.9 (5.82, 9.97)	7.71 (6.04, 9.37)	9.83 (7.88, 11.77)	−3.23 (−5.62, −0.83)	2.12 (0.36, 3.88)	2.61 (0.58, 4.64)
P trend income	0.14			< 0.01		
Education SII (CI)	11.7 (9.28, 14.12)	11.69 (9.94, 13.43)	10.71 (8.75, 12.68)	20.9 (18.36, 23.43)	14.00 (12.21, 15.78)	12.9 (10.95, 14.84)
P trend education	0.25			< 0.01		
Alcohol Use						
Income SII (CI)	−0.09 (−2.43, 2.24)	−2.64 (−4.59, 0.7)	3.08 (0.93, 5.23)	− 0.73 (−1.96, 0.49)	−0.15 (−1.29, 0.99)	−0.87 (− 2.05, 0.31)
P trend income	0.48			0.34		
Education SII (CI)	2.24 (1.16, 4.31)	−3.84 (−6.06, −1.61)	00.81 (−1.39, 3.01)	−0.97 (− 2.4, 0.47)	−1.13 (− 2.33, − 0.06)	−0.25 (− 1.01, 1.51)
P trend education	0.92			0.07		
Physical Inactivity						
Income SII (CI)	9.36 (7.00, 11.72)	10.97 (9.03, 12.91)	12.36 (9.84, 14.89)	10.67 (8.47, 12.87)	13.55 (11.72, 15.37)	12.06 (9.84, 14.28)
P trend income	0.04			0.43		
Education SII (CI)	10.89 (8.32, 13.46)	12.48 (10.4, 14.57)	7.83 (5.21, 10.45)	10.3 (7.98, 12.62)	13.97 (11.98, 15.96)	10.72 (8.28, 13.16)
P trend education	0.47			0.21		

^1^All estimates are age-adjusted

#### Smoking

The prevalence of smoking decreased between each survey for both sexes. Men appeared to experience larger income inequalities compared with women. Among women, the income inequality in 2006 favoured the low-income group, while the remaining inequalities favoured the high-income group. The income inequalities significantly in smoking increased over time for women but not for men. In contrast, women appeared to demonstrate larger educational inequalities compared with men. Women, but not men, experienced a significant decrease in inequalities over time. Women experienced larger educational inequalities compared with income, while the magnitude remained similar among men.

#### Alcohol

The prevalence of risky alcohol consumption was stable over the years. Due to the low prevalence of risky alcohol consumption, all inequalities appeared small. Income inequalities were only significant for men in 2014, while no income inequalities were significant for women. In both sexes, the inequalities in risky alcohol consumption did not change significantly over time. Education inequalities for men favoured the highly educated in 2006, but changed direction and favoured the lower educated in 2010. Similarly, for women the significant estimate in 2010 favoured the lower educated. Men appeared to experience larger education inequalities compared with women. Neither men’s nor women’s education inequalities changed significantly over time in risky alcohol consumption. Education inequalities appeared larger compared with income in men, while women only experienced statistically significant results in education.

#### Physical inactivity

The prevalence of a sedentary lifestyle remained stable over the years for both sexes. Magnitudes of income inequalities appeared similar for both sexes, significantly increasing among men over time. Likewise, the magnitude of education inequalities in sedentary lifestyle appeared similar, but no significant changes over time by either sex were observed. In both sexes, income and education magnitudes appeared to be similar.

## Discussion

Using an ‘outcome-wide’ approach, the results painted a complex picture of both increasing and decreasing health inequalities over time and with specific patterns being contingent on health outcome, socioeconomic indicator and sex. Specifically, income inequalities increased for psychological distress and physical inactivity in men as well as for poor SRH, overweight, hypertension, long-term illness, and smoking among women. No educational inequalities increased among men, but these increased for hypertension, long-term illness, and stress (the latter favouring the lower educated) in women. The only instance of decreasing income inequalities was seen for long-term illness in men, while education inequalities decreased for long-term illness in men and poor SRH, poor SRDH, and smoking in women. The remainder of the trends did not significantly change. Due to the large scope of the study and the complexity of the results, we have chosen to highlight some interesting aspects of the findings below. We discuss them primarily in relation to previous Swedish research and provide examples for illustration

### Differences in health inequalities by income and education

When observing numerical differences by income and education within each sex, the magnitude of health inequalities was larger for income compared with education in both men and women. Such variations, which suggest that the results depend on the choice of socioeconomic indicator, have been documented previously in the Swedish literature. Specifically, studies have focused on outcomes such as, for example, poor SRH [28] psychological resources [29], and chronic obstructive pulmonary disease [30], with the authors highlighting the importance of the socioeconomic indicator used. The variation dependent on the choice of socioeconomic indicator is an important finding because official Swedish reports tend to monitor inequalities mostly by education [13, 31]. This means that fluctuations resulting from income are consequently overlooked. The Public Health Agency of Sweden, for example, has until now reported only on educational inequalities in psychological distress, thus not capturing variations in the outcome between income groups [32]. However, the latest report accentuates that health inequalities are different depending on the indicator used [32].

### Variations in socioeconomic health inequalities between women and men

When looking at the findings from a sex perspective, the results indicate that education and income inequalities among men appear mainly stagnant, whereas inequalities among women tend to fluctuate. Furthermore, the results suggest that men experience a higher frequency of poor health, but the inequalities appear similar because there were no apparent numerical sex differences in the overall magnitude. This suggests that while men might experience worse health overall compared with women, the levels of absolute socioeconomic inequality appear similar between sexes. Similar to our findings, other Swedish research has documented differences in socioeconomic inequalities in BMI [33–35], cardiovascular morbidity [36], and poor SRH [37] between women and men.

### Disparate developments of socioeconomic health inequalities

Building on variations depending on the socioeconomic indicator used, the results from this study indicate that some health inequalities move in different directions, thereby further stressing the importance of choice of measure. For example, inequalities in poor SRH and smoking increased by income, but decreased by education. This is in contrast with a study [38] which reports that educational inequalities in poor SRH increased among women in Sweden. Furthermore, a noteworthy finding from the current study is the change of direction in smoking, where inequalities favoured the low-income group in 2006 (SII = − 3.23) and the high-income group in 2010 (SII = 2.61). This occurred because the richest quintile reduced their smoking and the poorest increased theirs. This finding stands in contrast with two Swedish studies reporting that inequalities in smoking appear to be increasing between education groups [39, 40].

### Reverse socioeconomic inequalities in health

The results from the current study suggest that some health outcomes are more prevalent among the highly educated or wealthy, which is counterintuitive to the common hypothesis that health status is more or less universally better among the richer and more educated [2]. In our study, two health outcomes followed such a pattern. The first was stress conditioned on education, for men and women, and the other was overweight conditioned on income among men. This finding is partly in line with a previous study which reported that BMI increased more in the higher rather than the lower socioeconomic groups in Sweden [33]. However, it contrasts with another Swedish study which concluded that obesity inequality was pro-rich among women and not statistically significant among men [41]. Nonetheless, reverse socioeconomic inequalities in Sweden, favouring the lower socioeconomic group, have also been found in ischemic stroke [42], brain tumour [43], food- and water-born infections [44], and snus use among female smokers [40].

### Socioeconomic inequalities across the different health dimensions

The results from this study further demonstrate interesting differences in socioeconomic inequalities between the four health dimensions: general health, physical health, mental health, and lifestyle habits. Overall, when looking across the sexes and the two socioeconomic indicators income and education, the general health dimension yielded the largest numerical inequality and mental health the lowest. Splitting the dimensions by income and education, the physical health income dimension resulted in the largest numerical inequality and mental health education dimension the lowest. When viewing men and women separately, men experienced the largest numerical inequality in general health and women in physical health. Both sexes experienced the smallest numerical inequality in mental health. Considering that few studies to date have utilised an outcome-wide approach, we have been unable to assess the extent to which these findings correspond to or contrast with previous research.

### Methodological considerations

The methodological strengths of this study include a large, age-diverse, population-based, random sample and the combination of survey data with linked socioeconomic information from high-quality total population Swedish registers. The use of an outcome-wide approach, which prevents us from finding isolated results for specific health outcomes [16] as well as the focus on absolute inequalities, which may be of greater public health relevance than the more commonly reported relative inequalities [45], are also strengths of this research. Despite this, the current study has some inherent limitations.

The data is cross-sectional with a participation rate around 50%, which may limit the generalizability of, and means that selection bias might have affected, the results. However, unfortunately the magnitude and direction of this bias could not be assessed due to the lack of available sociodemographic information on the population under study. However, underrepresentation with regard to education or income, for example, would not necessarily lead to incorrect estimates since they describe associations rather than just prevalence. It is nevertheless likely that more disadvantaged groups such as those with very low incomes and serious health problems mental disorders, may be underrepresented. Since the influence of this bias is ultimately unknown, the results should be interpreted with caution. Moreover, occupation was not included as a socioeconomic indicator and outcomes were dichotomized which could have resulted in a loss of information. In addition, the presence of only three observation points across 8 years and our estimation of linear trends may have been inadequate to capture patterns of socioeconomic health inequalities in the data. Due to these limitations, the results should be interpreted with caution.

## Conclusions and recommendations

Several interesting conclusions can be drawn from this study. Firstly, the findings demonstrate how patterns of socioeconomic inequalities in health may not be consistent, but instead vary depending on the health outcome and socioeconomic indicator studied, as well as between men and women. This disparate finding suggests that generalised and universal statements about the development of socioeconomic health inequalities in Sweden may be too simplistic and potentially misleading. Secondly, despite the complexity of socioeconomic inequalities in health shown in this study, the overall findings suggest that disparities exist, remain, and tend to increase. This result highlights the importance of developing interventions especially directed towards reducing the inequalities. Thirdly, the trends of socioeconomic inequalities in health appeared more stagnant in men while fluctuating in women, thereby indicating that policy makers need to apply a gender perspective when prioritising and implementing policies. Fourthly, socioeconomic inequalities in health by income generally appeared to be of greater magnitude than the corresponding disparities by education. This stresses the important role of income as potential generator of health inequalities and the need for policies targeting the income gap*.* Lastly, the inequalities varied between the different dimensions of health with aspects of general health (men) and physical health (women), especially with regard to income, showing the largest numerical differences. This finding highlights the importance of integrating a gender approach into the implementation of policies targeting the reduction of socioeconomic inequalities in specific gender health outcomes.

Because this approach gives a more nuanced picture of health inequalities, demonstrating differences conditioned on socioeconomic indicators and health dimensions, it can be of relevance to policy-makers by assisting in the prioritisation of public health recommendations.

## Data Availability

Access to the data used in the current study is managed by the register holders, the respective County Councils, and data are as such not publicly available.

## Abbreviations

BMI: Body mass index
GHQ-12: The General Health Questionnaire 12
OECD: The Organisation for Economic Co-operation and Development
SII: Slope index of inequality
SRDH: Self-rated dental health
SRH: Self-rated health
UK: United Kingdom
US: United States

## References

[1] SOU (2017). Nästa steg på vägen mot en mer jämlik hälsa - Förslag för ett långsiktigt arbete för en god och jämlik hälsa [The next step was towards a more equal health].

[2] Marmot M, Allen J, Bell R, Bloomer E, Goldblatt P (2012). WHO European review of social determinants of health and the health divide. Lancet..

[3] CSDH (2008). Closing the gap in a generation: health equity through action on the social determinants of health. Final report of the commission on social determinants of health.

[4] Kawachi I, Subramanian SV, Almeida-Filho N (2002). A glossary for health inequalities. J Epidemiol Community Health.

[5] Howel D, Stamp E, Chadwick TJ, Adamson AJ, White M (2013). Are social inequalities widening in generalised and abdominal obesity and overweight among English adults?. PLoS One.

[6] Tod E, Bromley C, Millard AD, Boyd A, Mackie P, McCartney G (2017). Obesity in Scotland: a persistent inequality. Int J Equity Health.

[7] Fors S, Thorslund M (2015). Enduring inequality: educational disparities in health among the oldest old in Sweden 1992-2011. Int J Public Health.

[8] Barr B, Kinderman P, Whitehead M (2015). Trends in mental health inequalities in England during a period of recession, austerity and welfare reform 2004 to 2013. Soc Sci Med.

[9] Bleich SN, Jarlenski MP, Bell CN, LaVeist TA (2012). Health inequalities: trends, progress, and policy. Annu Rev Public Health.

[10] Brown K, Nevitte A, Szeto B, Nandi A (2015). Growing social inequality in the prevalence of type 2 diabetes in Canada, 2004-2012. Can J Public Health.

[11] Jacquet E, Robert S, Chauvin P, Menvielle G, Melchior M, Ibanez G (2018). Social inequalities in health and mental health in France. The results of a 2010 population-based survey in Paris Metropolitan Area. PLoS One.

[12] Krieger N, Williams DR, Moss NE (1997). Measuring social class in US public health research: concepts, methodologies, and guidelines. Annu Rev Public Health.

[13] Folkhälsomyndigheten. Folkhälsans utveckling: Årsrapport 2019: Solna/Östersund: Folkhälsomyndigheten; 2019.

[14] SOU (2016). Det handlar om jämlik hälsa – Utgångspunkter för Kommissionens vidare arbete [It is about equity in health - the starting points of the Commission's further work]. specifu ed.

[15] Celeste RK, Fritzell J (2018). Do socioeconomic inequalities in pain, psychological distress and oral health increase or decrease over the life course? Evidence from Sweden over 43 years of follow-up. J Epidemiol Community Health.

[16] VanderWeele TJ (2017). Outcome-wide epidemiology. Epidemiology.

[17] Sweden S (2014). Teknisk Rapport: En beskrivning av genomförande och metoder - “Hälsa på lika villkor” Västerbotten [Technical Report: A description of implementation and methods - "Health on equal terms" Västerbotten].

[18] Goldberg DP, Gater R, Sartorius N, Ustun TB, Piccinelli M, Gureje O (1997). The validity of two versions of the GHQ in the WHO study of mental illness in general health care. Psychol Med.

[19] Lundin A, Hallgren M, Theobald H, Hellgren C, Torgén M (2016). Validity of the 12-item version of the general health questionnaire in detecting depression in the general population. Public Health.

[20] Schneider MC, Castillo-Salgado C, Bacallao J, Loyola E, Mujica OJ, Vidaurre M (2005). Summary of indicators most used for the measurement of the health inequalities. Epidemiol Bull.

[21] Oaks M, Kaufman J (2006). Methods in social epidemiology.

[22] Ernstsen L, Strand BH, Nilsen SM, Espnes GA, Krokstad S (2012). Trends in absolute and relative educational inequalities in four modifiable ischaemic heart disease risk factors: repeated cross-sectional surveys from the Nord-Trondelag health study (HUNT) 1984-2008. BMC Public Health.

[23] Mackenbach JP, Kunst AE (1997). Measuring the magnitude of socio-economic inequalities in health: an overview of available measures illustrated with two examples from Europe. Soc Sci Med.

[24] Regidor E (2004). Measures of health inequalities: part 2. J Epidemiol Community Health.

[25] Szilcz M, Mosquera PA, Sebastian MS, Gustafsson PE (2018). Time trends in absolute and relative socioeconomic inequalities in leisure time physical inactivity in northern Sweden. Scand J Public Health.

[26] Cherepanov D, Palta M, Fryback DG, Robert SA, Hays RD, Kaplan RM (2011). Gender differences in multiple underlying dimensions of health-related quality of life are associated with sociodemographic and socioeconomic status. Med Care.

[27] Vari R, Scazzocchio B, D'Amore A, Giovannini C, Gessani S, Masella R (2016). Gender-related differences in lifestyle may affect health status. Ann Ist Super Sanita.

[28] Ahlborg M, Svedberg P, Nyholm M, Morgan A, Nygren JM (2017). Socioeconomic inequalities in health among Swedish adolescents - adding the subjective perspective. BMC Public Health.

[29] Festin K, Thomas K, Ekberg J, Kristenson M (2017). Choice of measure matters: a study of the relationship between socioeconomic status and psychosocial resources in a middle-aged normal population. PLoS One.

[30] Axelsson Fisk S, Merlo J (2017). Absolute rather than relative income is a better socioeconomic predictor of chronic obstructive pulmonary disease in Swedish adults. Int J Equity Health.

[31] Socialdepartementet (2015). En kommission för jämlik hälsa.

[32] Folkhälsomyndigheten. Ojämlikheter i psykisk hälsa: Kunskapssammanställning: Solna/Östersund: Folkhälsomyndigheten; 2019.

[33] Al-Emrani F, Stafstrom M, Ostergren PO (2013). The influences of childhood and adult socioeconomic position on body mass index: a longitudinal Swedish cohort study. Scand J Public Health.

[34] Padyab M, Norberg M (2014). Socioeconomic inequalities and body mass index in Vasterbotten County, Sweden: a longitudinal study of life course influences over two decades. Int J Equity Health.

[35] Gustafsson PE, Persson M, Hammarstrom A (2012). Socio-economic disadvantage and body mass over the life course in women and men: results from the northern Swedish cohort. Eur J Pub Health.

[36] Waenerlund AK, Mosquera PA, Gustafsson PE, San SM. Trends in educational and income inequalities in cardiovascular morbidity in middle age in northern Sweden 1993-2010. Scand J Public Health. 2018;1403494818790406. 10.1177/1403494818790406 30113264

[37] Molarius A, Berglund K, Eriksson C, Lambe M, Nordstrom E, Eriksson HG (2007). Socioeconomic conditions, lifestyle factors, and self-rated health among men and women in Sweden. Eur J Pub Health.

[38] Copeland A, Bambra C, Nylen L, Kasim A, Riva M, Curtis S (2015). All in it together? The effects of recession on population health and health inequalities in England and Sweden, 1991-2010. Int J Health Serv.

[39] Giskes K, Kunst AE, Benach J, Borrell C, Costa G, Dahl E (2005). Trends in smoking behaviour between 1985 and 2000 in nine European countries by education. J Epidemiol Community Health.

[40] Norberg M, Malmberg G, Ng N, Brostrom G (2011). Who is using snus? - time trends, socioeconomic and geographic characteristics of snus users in the ageing Swedish population. BMC Public Health.

[41] Ljungvall A, Gerdtham UG (2010). More equal but heavier: a longitudinal analysis of income-related obesity inequalities in an adult Swedish cohort. Soc Sci Med.

[42] Novak M, Toren K, Lappas G, Kok WG, Jern C, Wilhelmsen L (2013). Occupational status and incidences of ischemic and hemorrhagic stroke in Swedish men: a population-based 35-year prospective follow-up study. Eur J Epidemiol.

[43] Khanolkar AR, Ljung R, Talback M, Brooke HL, Carlsson S, Mathiesen T (2016). Socioeconomic position and the risk of brain tumour: a Swedish national population-based cohort study. J Epidemiol Community Health.

[44] Pini A, Stenbeck M, Galanis I, Kallberg H, Danis K, Tegnell A (2019). Socioeconomic disparities associated with 29 common infectious diseases in Sweden, 2005-14: an individually matched case-control study. Lancet Infect Dis.

[45] Mackenbach JP (2015). Should we aim to reduce relative or absolute inequalities in mortality?. Eur J Pub Health.

